# A new prediction model for overall survival of elderly patients with solitary bone plasmacytoma: A population-based study

**DOI:** 10.3389/fpubh.2022.954816

**Published:** 2022-09-13

**Authors:** Yingying Wu, Jiemin Wei, Shaomei Chen, Xiaozhu Liu, Junyi Cao

**Affiliations:** ^1^Department of Blood Transfusion, The Second Affiliated Hospital of Guangxi Medical University, Nanning, China; ^2^Department of Hematology, The Second Affiliated Hospital of Guangxi Medical University, Nanning, China; ^3^Department of Hematology, The First Affiliated Hospital of Guangxi Medical University, Nanning, China; ^4^Department of Cardiology, The Second Affiliated Hospital of Chongqing Medical University, Chongqing, China; ^5^Department of Medical Quality Control, The First People's Hospital of Zigong City, Zigong, China

**Keywords:** solitary bone plasmacytoma, elderly patients, overall survival, SEER, nomogram, online application

## Abstract

**Background:**

Comprehensive studies on the prognosis of solitary bone plasmacytoma (SPB) are lacking, especially in elderly patients with SPB. This study aims to establish a novel nomogram and risk stratification system to predict the overall survival (OS) of elderly patients with SPB.

**Methods:**

The data of elderly patients with SPB from 2000 to 2017 were identified in the SEER database. SPB patients were randomly assigned to the training set (*n* = 825) and validation set (*n* = 354). The Cox regression analysis was used to determine the independent risk factors for OS in elderly SPB patients. The nomogram was established and assessed by the area under the receiver operating curve (AUC), the consistency index (C-index), and the calibration plot. Patients were divided into low-, medium-, and high-risk groups based on the score of the nomogram. The Kaplan-Meier (K-M) curve was used to verify the differences in overall survival among the three groups.

**Result:**

A total of 1,179 elderly patients with SPB were included in the study. Age at diagnosis, prior cancer before SPB, marital status, radiotherapy, and chemotherapy were independent risk factors of OS. The AUC of the 3, 5, and 8-year OS in the training and validation sets were between 0.707 and 0.860. The C-index and calibration plot also indicated that the nomogram has great predictive accuracy and robustness. After risk stratification, patients in the high-risk group had the worst OS.

**Conclusion:**

A novel nomogram was built to predict the OS of elderly patients with SPB. It will help clinicians formulate more reasonable and personalized treatment strategies.

## Introduction

Solitary plasmacytoma (SP) is a malignant tumor caused by monoclonal proliferation of plasma cells, accounting for about 3–5% of all plasma cell neoplasms (1, 2). SP can be divided into extramedullary plasmacytoma (EMP) and solitary bone plasmacytoma (SBP). SPB accounts for 60–70% of SP, mainly in the red marrow-containing bone, especially vertebrae and femurs (3, 4). SPB patients may experience bone pain, neurological symptoms, and pathological fractures, but lack multiple myelomas (MM) characteristics such as multiple lytic bone lesions, hypercalcemia, and renal insufficiency (5).

Studies have reported that older age, the primary site of the tumor, developing MM, histologic grade, treatment methods, and posttreatment persistent M protein were significant prognostic factors of SPB (6–12). Among them, age plays an important role in the prognosis of SPB patients. Studies have showed that age > 60 years was an important risk factor for the worse OS (13, 14) and progression to MM (14) in SPB patients. Similarly, older age (≥65 years) is significantly associated with the worse OS compared to younger SPB patients (1). Elderly SPB patients are more likely to receive palliative care rather than cure treatment. And older patients are more likely to be intolerant of radical radiation therapy than their younger counterparts (15). Meanwhile, elderly patients are more likely to be frail or have comorbidity. These factors may affect the survival of SPB patients. Therefore, it has a clinically important role in predicting the survival of elderly SPB patients.

Therefore, based on the SEER database, we collect data from a large number of patients to develop a survival prediction nomogram and a risk-stratifying system that can dynamically predict the long-term survival of elderly SPB.

## Methods

### Patient selection

**Figure 1:**
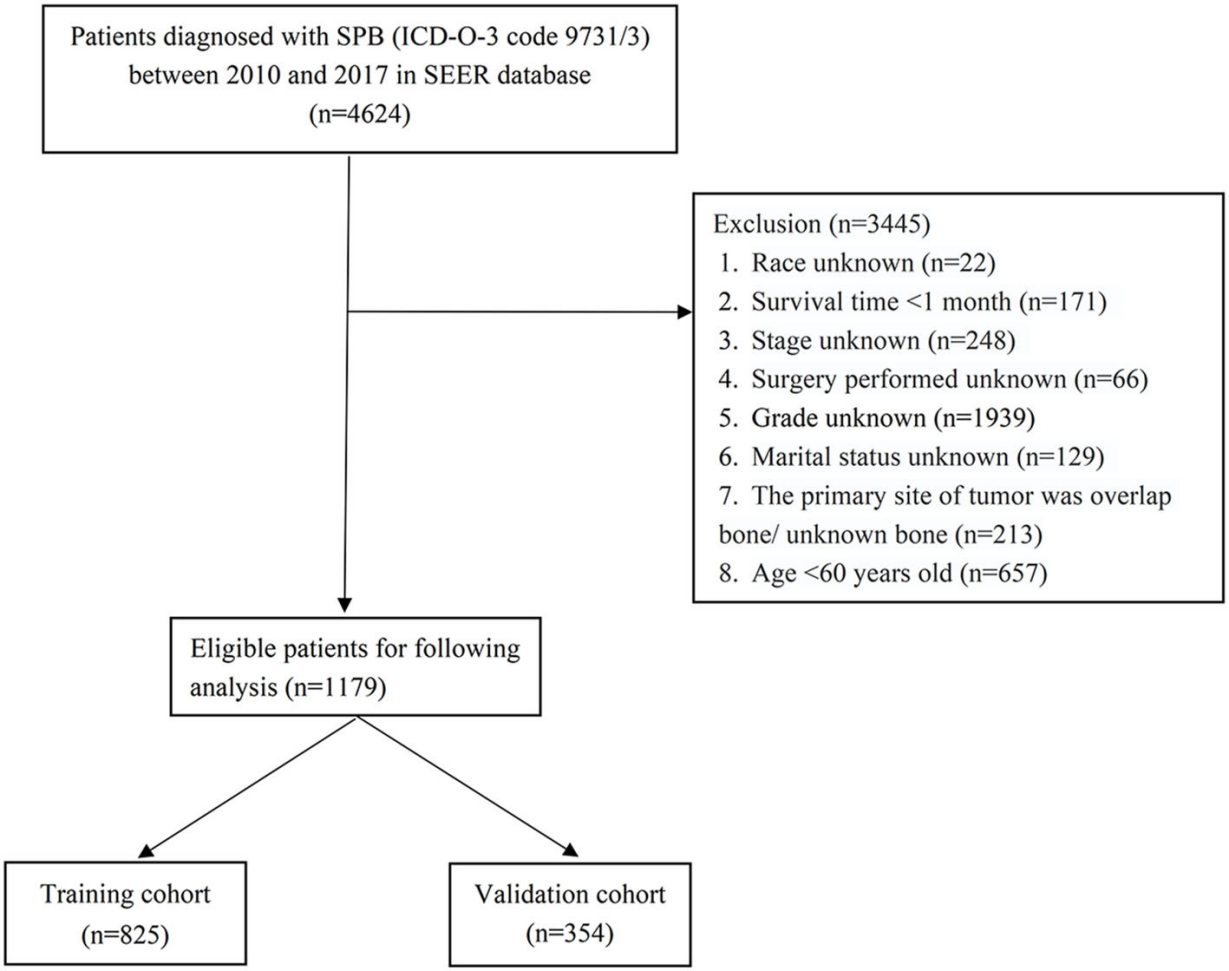
The flowchart of including and dividing patients.

All data in this study were obtained through the SEER^*^Stat software version 8.3.9. In the SEER database, subjects of SPB were identified by International Classification of Tumor Diseases, Third Edition (ICD-O-3) histology code 9731/3. Elderly Patients (≥60 years old) with SPB between 2000 and 2017 were included in our study. The individualized data we extracted from the SEER database included age at diagnosis, sex, tumor stage, age, race, sex, marital status, year of diagnosis, prior cancer before SPB, and treatment (radiotherapy, chemotherapy, and surgery), vital status, and survival time. Patients with incomplete individualized data were not included in this study. Also, patients diagnosed with SPB on death certificates or at autopsy were excluded from this study. The last follow-up day was December 31, 2018. The flow chart of patient screening is shown in Figure 1.

### Development of the nomogram

The elderly patients with SPB were randomly divided into a training set and validation set at a ratio of 7:3. Display and compare variables between training sets and validation sets. Classification variables were presented regarding the number of cases and percentage, and the chi-square test was used to compare groups. The Cox regression analysis was performed in the training set to identify the independent prognostic factors for OS in SPB patients. Candidate variables with a *p***-value < 0.25 on univariate analysis** were included in the multivariable model. Variables of *p*-value < 0.05 in the multivariate model were considered significantly related to OS. Based on these independent prognostic factors, a nomogram of OS was built. The receiver operating characteristic (ROC) curves and their corresponding AUC were generated to assess the discrimination of the nomogram. The calibration curves were used to measure the degree of agreement between the predicted probabilities of the model and the actual results. A risk stratification system was established. Based on the patient's total risk score, SPB patients were accurately divided into low, medium, and high-risk groups through X-tile software. The Kaplan-Meier curves were used to verify differences in OS among these risk groups.

### Statistical analysis

Statistical analysis was performed in R version 4.1.1 and SPSS statistics 24. A two-sided *P* < 0.05 was considered statistically significant.

### Ethics statement

The data extraction complies with the SEER database usage agreement. The data in the SEER database is public and does not require the patient's informed consent. Our study was exempt from review by the Ethics Committee of the Second Affiliated Hospital of Guangxi Medical University. All methods are carried out by relevant guidelines and regulations.

## Results

### Patient characteristics

1179 elderly patients with SPB were included in this study. in the whole cohort, 81.1% of patients were white, and 60.3% were male. The median age of SPB was 71.0 years old. 27.1% of patients had prior tumor before SPB Diagnosis. The most common tumor grade was the grade pre-B (97.3%). The most common primary sites of tumor were vertebrae bone (42.7%), followed by pelvis (17.6%), ribs/sternum/clavicle (16.6%), extremities (14.4%), and facial/skull bone (8.7%). As to treatment, 905 (76.8%) received radiotherapy, only 252 (21.4%) patients received surgery, and 236 (20.0%) received chemotherapy (Table 1). The patients were randomly divided into the training set (825 cases) and the validation set (354 cases). The characteristics of SPB patients are shown in Table 1.

**Table 1:** Baseline demographic and clinical characteristics of SPB patients.

**Variables**	**Total set**	**Training set**	**Validation set**	***P*-value**
	***N* = 1179**	***N* = 825**	***N* = 354**	
Age (years)	71.0 (8.3)	71.0 (8.2)	70.5 (8.6)	0.439
Sex				0.271
Female	468 (39.7%)	319 (38.7%)	149 (42.1%)	
Male	711 (60.3%)	506 (61.3%)	205 (57.9%)	
Race				0.901
Black	153 (13.0%)	105 (12.7%)	48 (13.6%)	
Other	70 (5.9%)	50 (6.1%)	20 (5.6%)	
White	956 (81.1%)	670 (81.2%)	286 (80.8%)	
Marriage				0.249
No	401 (34.0%)	272 (33.0%)	129 (36.4%)	
Yes	778 (66.0%)	553 (67.0%)	225 (63.6%)	
Primary cancer				0.374
Yes	860 (72.9%)	608 (73.7%)	252 (71.2%)	
No	319 (27.1%)	217 (26.3%)	102 (28.8%)	
Primary site				0.365
Vertebrae	504 (42.7%)	358 (43.4%)	146 (41.2%)	
Pelvis	207 (17.6%)	148 (17.9 %)	59 (16.7%)	
Ribs/Stenum/Clavicle	196 (16.6%)	142(17.2%)	54 (15.3%)	
Extremities	170 (14.4%)	112 (13.6%)	58 (16.4%)	
Facial/Skull Bone	102 (8.7%)	65 (7.9%)	37 (10.5%)	
Tumor grade				0.182
Pre-B cell	1147 (97.3%)	798 (96.7%)	349 (98.6%)	
Grade I	8 (0.7%)	7 (0.8%)	1 (0.3%)	
Grade II	4 (0.3%)	2 (0.2%)	2 (0.2%)	
Grade III	6 (0.5%)	6 (0.7%)	0 (0.0%)	
Grade IV	14 (1.2%)	12 (1.5%)	2 (0.6%)	
Chemotherapy				0.276
No/Unknown	943 (80.0%)	653 (79.2%)	290 (81.9%)	
Yes	236 (20.0%)	172 (20.8%)	64 (18.1%)	
Radiotherapy				0.913
No/Unknown	274 (23.2%)	191 (23.2%)	83 (23.4%)	
Yes	905 (76.8%)	634 (76.8%)	271 (76.6%)	
Surgery				0.235
No	927 (78.6%)	641 (77.7%)	286 (80.8%)	
Yes	252 (21.4%)	184 (22.3%)	68 (19.2%)	

SPB, Solitary bone plasmacytoma; Other American Indian/Alaska Native, Asian/Pacific Islander.

### OS and prognostic factors of the training set

In the training set, the median OS of SPB patients was 64 (1–209) months. And the 3, 5, and 8-year OS was 63.9%, 51.1%, and 37.2%, respectively. The Kaplan–Meier survival analyses were used to stratify patients according to their demographics and treatment patterns. Age at diagnosis, marital status, prior cancer, tumor grade, radiotherapy, surgery, and chemotherapy were risk factors for OS. The above factors were included in the multivariate cox regression analysis. The results showed that age at diagnosis, marital status, prior cancer, radiotherapy, and chemotherapy were considered independent prognosis factors (Table 2).

**Table 2:** Univariate and multivariate analysis of OS for SPB patients in the training set.

**Variables**	**Univariate analysis**		**Multivariate analysis**	
	**HR (95% Cl)**	***p*-value**	**HR (95% Cl)**	***p*-value**
Age (years)	1.073 (1.060–1.086)	<0.001	1.069 (1.055–1.083)	<0.001
Sex		0.686		
Female	Reference			
Male	0.96 (0.781–1.177)			
Race		0.298		
Black	Reference			
Other	1.011 (0.630–1.623)	0.964		
White	0.825 (0.619–1.100)	0.190		
Marrige		0.001		<0.001
No	Reference		Reference	
Yes	0.739 (0.615–0.887)		0.619 (0.503–0.763)	
Primary site		0.930		
Vertebrae	Reference			
Pelvis	0.897 (0.671–1.198)	0.461		
Ribs/Stenum/Clavicle	1.000 (0.753–1.327)	0.998		
Extremities	0.961 (0.706–1.308)	0.800		
Facial/Skull Bone	1.071 (0.734–1.563)	0.723		
Tumor grade		0.117		0.152
Pre-B cell	Reference		Reference	
Grade I	1.679 (0.325–8.681)	0.536	1.298 (0.373–4.513)	0.681
Grade II	3.255 (0.985–10.754)	0.053	1.149 (0.371–3.560)	0.810
Grade III	1.888 (0.631–5.654)	0.256	2.105 (0.747–5.935)	0.159
Grade IV	1.207 (0.496–2.940)	0.678	0.878 (0.408–1.888)	0.739
Primary cancer		0.035		0.015
Yes	Reference		Reference	
No	1.265 (1.017–1.574)		1.321 (1.057–1.652)	
Chemotherapy		0.025		0.004
No/Unknown	Reference		Reference	
Yes	1.307 (1.035–1.652)		1.423 (1.122–1.804)	
Radiotherapy		<0.001		0.003
No/Unknown	Reference		Reference	
Yes	0.575 (0.463–0.714)		0.712 (0.571–0.888)	
Surgery		0.01		0.391
No	Reference		Reference	
Yes	0.725 (0.562–0.936)		0.893 (0.689–1.157)	

OS, Overall survival; SPB, Solitary bone plasmacytoma; HR, Hazard ratio; Other American Indian/Alaska Native, Asian/Pacific Islander.

### Nomogram of the OS

**Figure 2:**
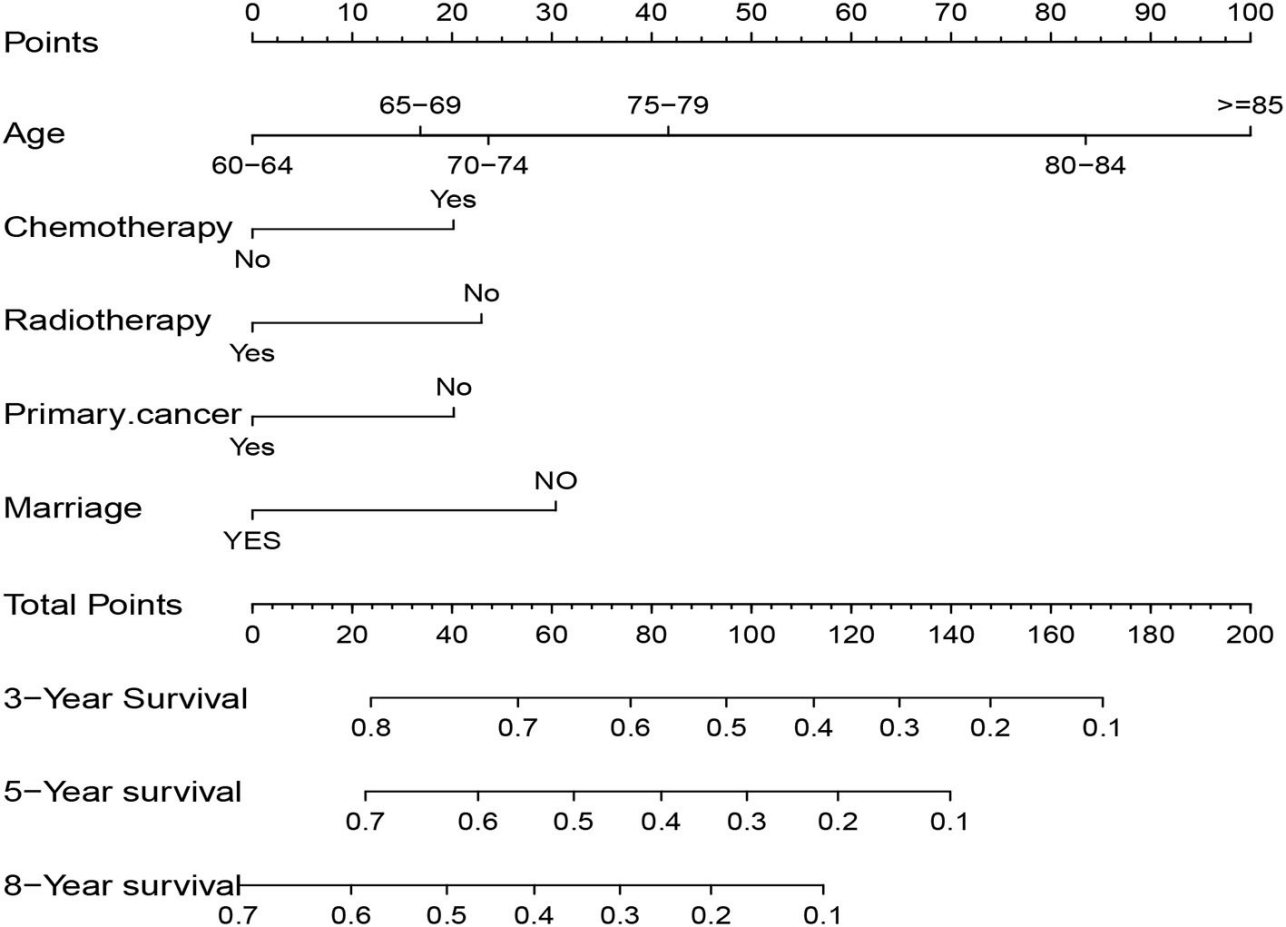
Nomogram to predict 3, 5, and 8-year overall survival in elderly patients with solitary bone plasmacytoma.

**Figure 3:**
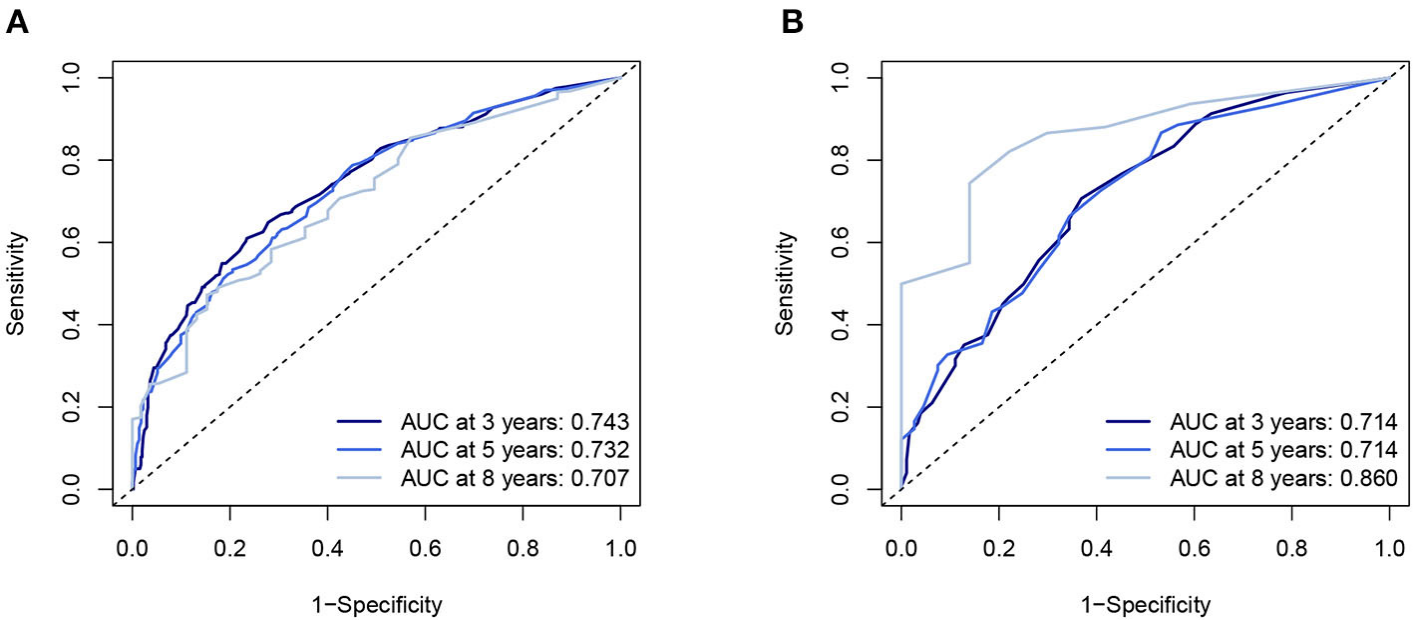
The AUC of nomogram of 3-, 5-, and 8-year in the training set **(A)** and validation set **(B)**. AUC, Receiver operating curve.

**Figure 4:**
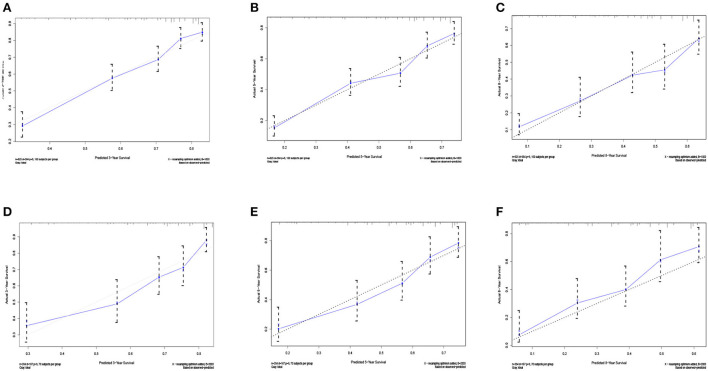
The calibration curves for predictions of overall survival in the training set **(A–C)** and validation set **(D–F)** at 3, 5, and 8-year.

Five independent prognostic factors were included to construct the nomogram for OS (Figure 2). The ROC curves showed that the AUC of the 3, 5, and 8-year OS were 0.743, 0.732, and 0.707 in the training set and 0.714, 0.714, and 0.860 in the validation set (Figure 3). The C-index of the training set and validation set were 0.688 (95% CI: 0.660–0.718) and 0.691 (95% CI: 0.650–0.732), respectively. The calibration curves showed great agreement between the predictions and actual outcomes for 3, 5, and 8-year survival (Figure 4).

### Risk stratification based on prognostic nomogram

**Figure 5:**
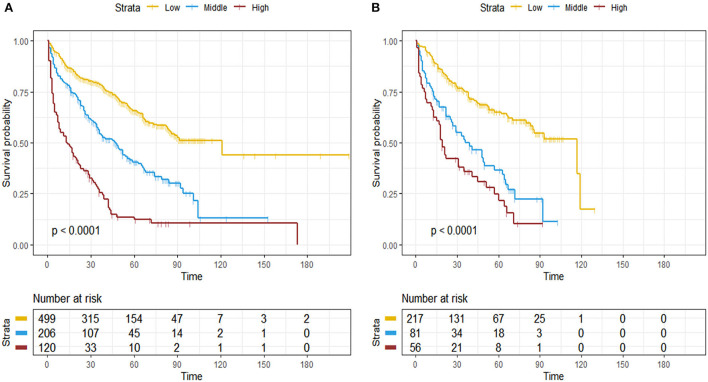
Kaplan-Meier survival curves for the training set **(A)** and validation set **(B)**.

The total score of patients was calculated according to the nomogram. The best cut-off point of the total score was determined by X-tile software. The cut-off points are 63 and 106. And we designated a total score of < 63 as the low-risk group, between 63 and 106 as the medium-risk group, and a score > 106 as the high-risk group. The survival curves showed that the survival curves of the three risk groups were significantly different (*P* < 0.001), whether in the training or validation set. Elderly patients with SPB in the high-risk group have the worst OS, while patients in the low-risk group have the best OS (Figure 5).

### Online application for OS prediction

We have developed a user-friendly online application to predict the OS of elderly SPB patients, which can be accessed at https://yingyingwu.shinyapps.io/DynNomapp/. Enter the patient's personal and clinical characteristics; we can immediately predict the patient's survival probability. In short, this calculating tool is convenient and friendly to both patients and physicians.

## Discussion

The SEER database covers a great number of cancer cases with complete follow-up. It is often used in combination with nomograms to predict the survival of cancers (16, 17). We included 1,179 elderly SPB patients to analyze clinical and demographic sociology-related prognostic factors. Then we built a nomogram to predict the OS of elderly SPB patients. The nomogram was based on five independent risk factors: age, marital status, prior cancer, radiotherapy, and chemotherapy. And with the increase of age, the immune system's ability continues to decline, which may cause tumor deterioration or serious treatment complications, reducing the patient's survival time. Older patients often have more comorbidities such as diabetes, cardiovascular and cerebrovascular diseases, and other cancers than younger patients. It may directly have a negative impact on survival or affect treatment tolerance (18). Also, in this study, prior cancer before SPB and older age are the negative factors in elderly patients with SPB.

Marital status is a significant prognostic factor for many cancers (19–21). Compared with unmarried or divorced patients, married patients may get better financial, emotional support, and life care from their partners or family members. Therefore, it may be associated with a better prognosis. Similarly, we found that married was a favorable prognostic factor for OS in SPB patients.

Radiotherapy, surgery, and systematic chemotherapy are the commonly used treatments for SPB. The guidelines recommend that the standard treatment of SPB is local radiotherapy (22, 23). Local radiotherapy can provide good local control and survival for SPB patients (11, 24, 25). And our study reaffirmed the benefits of radiotherapy for SPB patients. We found that radiotherapy was the primary treatment, and 78.8% of patients received radiotherapy. Radiotherapy can significantly improve the OS of SPB patients.

Guidelines recommend that surgery may be considered when SPB patients have a structural imbalance or neurological damage caused by tumor (22). Our results suggested that surgery does not affect patient outcomes. Surgery can only relieve symptoms of SPB patients and improve their self-care ability, but it may not stop the progression of the SPB.

Currently, adjuvant chemotherapy is not recommended due to insufficient research support. Several previous multicenter studies have shown that chemotherapy does not benefit the survival of SPB patients (14, 26, 27). A recent survey by Khaled et al. shows that the addition of chemotherapy mainly based on bortezomib/dexamethasone or lenalidomide/dexamethasone improves multiple myeloma-free survival and PFS in patients with SPB (28). However, it was retrospective studies of a small sample, and additional adjuvant chemotherapy did not improve the survival of SPB in most previous studies. More evidence is needed in the future. In our study, 236 (20.0%) patients received chemotherapy. The results showed that chemotherapy is an adverse prognostic factor. The following reasons should be considered: chemotherapy is often used in patients who are intolerant to radiotherapy or have the potential risk of developing MM. The prognosis of those SPB patients is often poor. Besides, we must acknowledge that there is no detailed information about chemotherapy regimens in our data, and we cannot compare the effects of different chemotherapy regimens on survival. It may lead to biased final results.

However, some limitations of our research should be noted. Firstly, it was a retrospective study, and the potential selection bias was unavoidable. Secondly, since detailed treatment data such as chemotherapy and radiotherapy regimens cannot be obtained from the SEER database, the treatment effect cannot be further evaluated. Third, much clinical and pathological information, such as the extent of bone marrow involvement and the M protein in blood/urine cannot be obtained, which may lead to study bias.

Undeniably, it was a large population-based study, and its results were representative. Then, we constructed an effective nomogram to evaluate the OS of elderly SPB patients. The excellent performance of the nomogram has been confirmed by ROC curves, calibration curves, and decision curve analysis.

## Conclusion

In summary, the novel nomogram and risk-stratifying system could effectively predict long-term OS in elderly patients with SPB and identify high-risk patients. It is of great significance to improve the prognosis of patients.

## Data availability statement

The original contributions presented in the study are included in the article/supplementary material, further inquiries can be directed to the corresponding authors.

## Author contributions

YW and JC designed the main study. JW and SC collected and analyzed the data. YW and XL made contributions to the drafting of the manuscript. JC contributed substantially to the revision of the manuscript. All authors have read and approved the final draft of the manuscript.

## Conflict of interest

The authors declare that the research was conducted in the absence of any commercial or financial relationships that could be construed as a potential conflict of interest.

## Publisher's note

All claims expressed in this article are solely those of the authors and do not necessarily represent those of their affiliated organizations, or those of the publisher, the editors and the reviewers. Any product that may be evaluated in this article, or claim that may be made by its manufacturer, is not guaranteed or endorsed by the publisher.
